# A diabetes-like biochemical and behavioural phenotype of *Drosophila* induced by predator stress

**DOI:** 10.1098/rspb.2023.0442

**Published:** 2023-07-12

**Authors:** Tatjana Krama, Diana Bahhir, Liina Ots, Sergejs Popovs, Vadims Bartkevičs, Iveta Pugajeva, Ronalds Krams, Enno Merivee, Anne Must, Markus J. Rantala, Indrikis Krams, Priit Jõers

**Affiliations:** ^1^ Department of Biotechnology, Daugavpils University, 5401 Daugavpils, Latvia; ^2^ Chair of Plant Health, Institute of Agricultural and Environmental Sciences, Estonian University of Life Sciences, 51014 Tartu, Estonia; ^3^ Institute of Molecular and Cell Biology, University of Tartu, EE-51010, Tartu, Estonia; ^4^ Institute of Food Safety, Animal Health and Environment ‘BIOR’, Riga 1076, Latvia; ^5^ Department of Biology & Turku Brain and Mind Centre, University of Turku, 20014 Turku, Finland; ^6^ Department of Zoology and Animal Ecology, Faculty of Biology, University of Latvia, Riga 1004, Latvia; ^7^ Institute of Ecology and Earth Sciences, University of Tartu, 51010 Tartu, Estonia; ^8^ Latvian Biomedical Research and Study Centre, Riga 1067, Latvia

**Keywords:** glucose, predation, stress, diabetes, serotonin, *Drosophila melanogaster*

## Abstract

Predation can have both lethal and non-lethal effects on prey. The non-lethal effects of predation can instil changes in prey life history, behaviour, morphology and physiology, causing adaptive evolution. The chronic stress caused by sustained predation on prey is comparable to chronic stress conditions in humans. Conditions like anxiety, depression, and post-traumatic stress syndrome have also been implicated in the development of metabolic disorders such as obesity and diabetes. In this study, we found that predator stress induced during larval development in fruit flies *Drosophila melanogaster* impairs carbohydrate metabolism by systemic inhibition of Akt protein kinase, which is a central regulator of glucose uptake. However, *Drosophila* grown with predators survived better under direct spider predation in the adult phase. Administration of metformin and 5-hydroxytryptophan (5-HTP), a precursor of the neurotransmitter serotonin, reversed these effects. Our results demonstrate a direct link between predator stress and metabolic impairment, suggesting that a diabetes-like biochemical phenotype may be adaptive in terms of survival and reproductive success. We provide a novel animal model to explore the mechanisms responsible for the onset of these metabolic disorders, which are highly prevalent in human populations.

## 1. Introduction

Physiological, social and ecological factors can act as stressors capable of affecting the development of behavioural or biochemical phenotypes of organisms. Although stress responses often trigger adaptive physiological or behavioural changes by improving the survival of organisms, prolonged stress may harm the body by initiating life-threatening effects, impairing survival, and causing disease and death. Stress can aggravate various pathophysiological complications and metabolic changes in organisms [1]. Stress can also affect the nervous system of organisms and cause structural changes in different parts of their brains [2] through such processes as atrophy and decreased brain mass [3]. These effects may significantly impair memory, learning and cognition [4].

Mental disorders such as depression, anxiety and post-traumatic stress disorders (PTSD) are prevalent in human populations, affecting approximately 10% of people globally, a number that is steadily increasing [5]. The primary effects of metabolic disorders on quality of life are usually considered to be caused by adverse behavioural changes, leading to impaired emotional responses and loss of autonomy [6].

While several brain-specific metabolic alterations caused by these stress-related pathologies have been extensively studied, less is known about their effects on systemic metabolism [7,8]. This hypothetical connection, however, is increasingly coming to focus as numerous epidemiological studies have underlined the association of psychological conditions with dysfunctional glucose catabolization and insulin resistance [7]. Moreover, psychological stress has been implicated as a cause for the development of diabetes in initially healthy individuals [9–11]. These stress conditions do not, therefore, only impair cognitive functions and aggravate the clinical outcome of comorbid metabolic pathologies. Still, they are also potentially one of the factors increasing the growing prevalence of metabolic disorders. This view is supported by experimental rodent models, where chronic unpredictable mild stress and social defeat initiate metabolic dysregulation, leading to both peripheral and brain hyperglycaemia [12,13].

While mental stress induces numerous changes in both human and animal neuronal biochemistry and physiology [14–16], our understanding of the signalling pathway(s) connecting these alterations with general metabolism is fragmentary at best [17,18]. Much of the focus has been on the regulation of glucocorticoid hormone secretion released by the hypothalamic–pituitary–adrenal (HPA) axis [19]. These hormones control the systemic regulation of glucose utilization by inhibiting glucose uptake in muscle tissue and inducing gluconeogenesis in the liver from other molecules (e.g. branched-chain amino acids) [20–22]. This is believed to be an adaptation to conserve glucose for tasks such as the flight-or-fight response, increasing the odds of surviving an imminent attack [23,24]. However, prolonged activation of the HPA axis might become maladaptive, and its chronic upregulation is a well-defined consequence of psychological stress [25]. However, not all cohort studies demonstrate a connection between stress, glucocorticoids and metabolic dysregulation, suggesting that there are additional metabolic regulation pathways [26–28].

Here we test whether spider predation induces metabolic dysregulation in *Drosophila melanogaster* during larval development and whether the observed biochemical changes affect overall energy levels, locomotor activity, and survival of adult flies. We report that inducing predator stress in *D. melanogaster* causes systemic inhibition of Akt kinase, a central regulatory protein controlling glucose uptake in cells. At the organismal level, this leads to an impaired ability to metabolize glucose, suppressing glycolysis and shifting catabolism towards the utilization of fatty acids. Suppressing the consumption of carbohydrates has negative effects on locomotor activity and resistance to acute and chronic starvation. However, the findings of this study showed that predator-induced stress also resulted in increased survival in a predator-rich environment. Restoring normal metabolism either with serotonin supplementation or with metformin feeding negated the survival advantage. This indicates that metabolic reprogramming with long-term negative health effects may be adaptive by nature, sacrificing metabolic balance to enable escaping immediate death via, e.g. increased memory creation and, consequently, improved behavioural responses in the presence of direct threats to survival. Our work provides a novel understanding of how conditions similar to psychological stress can alter systemic energy catabolism and introduces a new animal model with exceptionally powerful genetics for future research on the topic.

## 2. Methods

### (a) *Drosophila* husbandry and food formulations

*Drosophila* flies were reared in incubators at 23 ± 1°C under a constant 12 : 12 h light–dark cycle. This study used the wild strains Oregon-R-modENCODE (no. 25211) and *w^1118^* of *D. melanogaster* obtained from the Bloomington Drosophila Stock Centre (IN, U.S.A.). The flies were isolated and sexed under carbon dioxide anaesthesia. To obtain *Drosophila* for this study, we placed 10 female and 5 male flies together to copulate and oviposit for 24 h in 24.5 × 95 mm vials (Genesee Scientific, San Diego, CA, USA). Each vial contained 18 ml of food. The food was prepared as a mixture of 500 ml water, 20 g dextrose, 15 g sucrose, 10 g brewer's yeast, 35 g cornmeal, 4.5 g agar and 12.5 ml 10% Tegosept (methyl-*p*-hydroxybenzoate) stock solution [29]. When required, metformin (Acros Organics, AC429720050) or 5-hydroxytryptophan (5-HTP) was added to the medium after cooling below 65°C, at concentrations of 20 mM and 5%, respectively.

The vials with eggs were placed horizontally on the floor of plastic jars (10 cm height × 12 cm diameter). Each jar in the experimental groups contained one pirate otter-spider (*Pirata piraticus*) collected throughout the spring/summer seasons. Flies remained together with spiders for their egg and larval stages [30]. The spiders were free to walk into the vials, where they often attacked and consumed *D. melanogaster* larvae.

Adult flies were collected within 5–7 h after the imaginal eclosion for biochemical analyses or used for behavioural assays within 2–3 days after eclosion. The flies used for biochemical investigation were frozen at −80°C.

### (b) Feeding experiments

To measure the rate of feeding, food supplemented with blue dye (Blue FCF dye, Acros Organics A0373695, ThermoFisher Scientific) was fed to flies. The amount was quantified spectrophotometrically from homogenate. For each experiment, 140 flies from the control condition and 140 from the predator-stress condition were placed in two separate standard food bottles, and allowed to recover overnight from CO_2_ exposure. On the next day, the flies were transferred without gas either to a new standard food or to food supplemented with 1% Blue FCF dye. After 1.5 h, 20 flies were collected and homogenized on ice by grinding in a mortar and pestle in 800 μl phosphate-buffered saline (PBS). Debris was pelleted at 10 000***g***_max_ for 10 min at 4°C, and 400 µl of each supernatant was transferred to 2 wells (200 µl each) of 96-well plates. Absorbance was measured at 650 nm, and values from lysates of flies kept on food without Blue FCF were used for background subtraction.

### (c) Starvation tolerance measurements

In the chronic starvation tolerance test, flies were kept on 1% agar in tubes containing 10 individuals. Survival was monitored every 3 h. Death was determined as the last activity time point from the final recorded activity for each fly. In the acute starvation tolerance test, flies were starved on deionized water-soaked filter paper in tubes containing 10 individuals. The moisture content of the paper was controlled by injecting water with a syringe once a day.

### (d) Survival of *Drosophila* under spider predation

To assess whether a diabetes-like phenotype has any adaptive value, we tested the survival of *Drosophila* under conditions of direct predation by spiders. We used 10 experimental and 10 control groups, each consisting of 10 male flies. We placed each group in a plastic container (20 cm width, 10 cm depth, 10 cm height) for 12 h during daylight time. Each jar contained one pirate otter-spider and one vial with *Drosophila* food (cornmeal, dextrose, sucrose, agar and yeast medium). We placed a layer of filter paper on the bottom of each container, and the top was covered by mash. The spiders were left without food for 12 h before the trials, while water was provided before and during the tests. Surviving flies were counted at the end of the experiment. Each otter-spider was used only once.

### (e) Behavioural assays

We used sterile Petri dishes moulded from clear polystyrene (60 × 15 mm; Flystuff, El Cajon, USA) as novel arenas to record individual flies' locomotor activity. Only one fly was aspirated into the arena for each test. The locomotor activity of six flies was recorded with the resolution of 1920 × 1080 pixels at 5 frames per second simultaneously by a video-tracking system using the Logitech HD Pro Webcam C920 (Logitech Inc., Newark, CA, USA), fixed at a height of 25 cm above the arenas, and the software Debut Video Capture (NCH Software, Greenwood Village, CA, USA). To shorten the experiment duration, two identical video-tracking systems were prepared, which allowed tracking of 12 flies simultaneously. The video-tracking course was 15 min. We calculated the flies’ average speed for each minute. The arenas were illuminated by reflected, diffused light from above by four MR 16 LED lamps (12 V, 6 W, 400 lm, 3000 K) located 0.9 m above the arenas. Illumination at the level of the arenas (3000 lux) was measured by a TES-1335 Digital Light Meter (TES Electrical Electronic Corporation, Taipei, Taiwan). All video recordings were made in the laboratory at between 21 and 22°C, and 35–40% relative humidity. Distance moved (start speed >0.20 mm s^−1^; stop speed <0.20 mm s^−1^) with the temporal bin width of 1 min as the most important locomotor activity parameter was extracted offline from the recorded video files using EthoVision XT Version 11 software (Noldus Information Technology, Wageningen, The Netherlands). The distances moved were used to calculate the speed, representing the integral values of distances and time.

### (f) Western analyses

Batches of 30 flies were homogenized with a pestle on ice in 300 µl of western lysis buffer (PBS with 1.5% Triton X-100) supplemented with protease and phosphatase inhibitor cocktails (Roche Complete Mini no. 11836170001 and PhosSTOP no. 04906845001) following the manufacturer's protocols. Lysates were incubated on ice for 15 min and then centrifuged at 13 000***g***_max_ for 15 min at 4°C to pellet debris. Supernatant protein concentrations were measured using the Bradford assay (Thermo no. 1856209), and 70 µg aliquots were loaded onto precast Bio-Rad Criterion AnyKD gradient gels. Gels were run in ProSieve EX running buffer (Lonza). Proteins were transferred to Amersham Protran nitrocellulose membrane (no. 10600020) in ProSieve EX transfer buffer (Lonza) at 35 V for 50 min in a BioRad Criterion Transfer chamber. Membranes were incubated in 5% BSA in 1× TBS/0.05% Tween for 1 h for blocking, after which they were incubated overnight at 4°C in the same buffer with primary antibodies. Antibodies and dilutions used were: Akt 1 : 5000 (Cell Signaling no. 9272), phospho-Akt 1 : 5000 (Cell Signaling no. 4054), ACC 1 : 5000 (Cell Signaling no. 3676), HRP-conjugated anti-rabbit 1 : 10 000 (PI-1000-1).

After washing membranes three times for 15 min with 1× TBS/0.05% Tween, they were incubated with anti-rabbit secondary antibody conjugated with horseradish for 1 h at ambient room temperature. After an additional three rounds of washing as before, results were visualized with the BioRad ChemiDoc XR detection system. For quantitation purposes, samples from control and predator-reared flies were run on the same gel with four individual biological replicates per group. When protein amount per lane was used for normalization, membranes were stained with Ponceau S solution (0.1% Ponceau S in 5% acetic acid), rinsed briefly with water, and documented using the BioRad ChemiDoc XR system. The signal was quantified, and the data were analysed with ImageQuant software. Western blots and corresponding Ponceau S-stained membranes used for quantifications are presented in electronic supplementary material, figure S1.

### (g) Metabolite analyses

For carbohydrate measurements, 10 flies were homogenized in 400 µl of PBS and incubated for 5 min at 70°C. A total of 40 μl of lysate was transferred to four separate Eppendorf tubes with additions of 1 U of amyloglucosidase from *Aspergillus niger* (Sigma, total glucose measurement), 2× PBS (free glucose and background measurement) and 5 mU of porcine kidney trehalase (Sigma T8778, trehalose measurement). All reactions were incubated for 2 h at 37°C, after which they were briefly centrifuged, and 30 µl of supernatant was transferred to 96-well microtitre plates. One hundred microlitres of Glucose Assay Reagent (Sigma G3293) was added to all reactions except for one PBS-treated lysate mixed with 100 µl of PBS to measure the background signal. Reactions were incubated at 37°C for 30 min, after which absorption was measured at 340 nm. Free glucose, glycogen and trehalose were calculated by subtracting relevant backgrounds from measured values. A glucose standard curve was generated using 1 to 20 µg of glucose (per well). The results were normalized against protein amount measured with Bradford assay (Thermo no. 1856209).

For triglyceride measurements, 10 flies were homogenized in 800 µl of PBS with 0.1% Tween 20 and incubated for 5 min at 70°C. Twenty microlitres of each lysate was transferred to three Eppendorf tubes with additions of 20 µl of Triglyceride Reagent (Sigma T2449, total glycerol measurement) and 2 × 20 µl of PBS (free glycerol and background measurement). All reactions were incubated for 30 min at 37°C, then briefly centrifuged, and 30 µl of supernatant was transferred to 96-well microtitre plates. One hundred microlitres of Free Glycerol Reagent (Sigma F6428) was added to all reactions except for one PBS-treated lysate mixed with 100 µl of PBS to measure the background. Reactions were incubated at 37°C for 5 min, after which absorption was measured at 540 nm. Triglycerides were calculated by subtracting free glycerol from total glycerol measurement. A glycerol standard curve was calculated using 0.5 to 3 µg of glycerol (per well). The results were normalized against protein amount measured with Bradford assay (Thermo no. 1856209).

ATP concentration was measured using the ATP Determination kit (ThermoFisher Scientific). Thirty flies were homogenized in ATP isolation buffer (6 M guanidine-HCl, 4 mM EDTA, 100 mM Tris/Cl pH 7.8) and snap-frozen in liquid nitrogen, followed by boiling for 5 min. Debris was pelleted by centrifugation at 10 000***g***_max_ for 10 min at 4°C. Five microlitres of a 12.5-fold diluted supernatant was added to 100 µl of ATP Reaction Mix (Thermo Fisher; formulated according to the manufacturer's recommendations), and values were recorded using a Tecan luminometer with Greiner polypropylene plates (no. 655207). The results were normalized against protein amount measured with Bradford assay (Thermo no. 1856209).

Pyruvate was measured using BioVision kit no. K709 according to the modified protocol provided by the manufacturer. For pyruvate measurements, 20 flies were homogenized in 200 µl Pyruvate Assay Buffer on ice and then centrifuged at 10 000***g***_max_ for 10 min at 4°C. Fifteen microlitres of supernatant was mixed with 35 µl of Pyruvate Assay Buffer in a well of the 96-well microtitre plate. Fifty microlitres of reaction mix (formulated according to the manufacturer's guidelines) was added to each well containing supernatant and incubated for 30 min at room temperature, after which absorption was measured at 570 nm. Parallel background reactions were performed by mixing supernatant with background mix, formulated according to the manufacturer's guidelines. The results were normalized against protein amount measured with Bradford assay (Thermo no. 1856209).

### (h) Respiration exchange ratio measurements

Respiration exchange ratio (RER) was calculated as the ratio of CO_2_ produced and O_2_ used by flies. O_2_ consumption in individual flies was measured by coulometric respirometry in a continuous O_2_-compensating system at constant temperature and humidity (23°C and 55% relative humidity). Flies were placed into measuring chambers, and measurements were begun when the flies stopped moving and the minimum value of gas exchange was reached. CO_2_ levels were determined using a LI-700 differential CO_2_/H_2_O analyser (LiCor, Lincoln, Nebraska, USA).

### (i) Statistics

All measures (except for locomotor activity) are averages of four to ten biological replicates, and individual values are marked with red dots on diagrams. Each individual biological replicate measurement of metabolites, feeding and quantification of proteins on western blots represents 10–30 flies per sample, depending on the assay (see above). For the locomotory speed measurements, 24 (control) or 16 (predator-reared) flies were used. For metabolite, protein, feeding, RER, locomotion, and survival measurements, *p*-values were calculated using two-tailed Student's *t*-tests. Error bars represent standard deviations. In the case of locomotor activity measurements, nonlinear regression of one phase decay model was used: $Y=(Y_{0}-\mathrm{baseline})e^{-KX}+\mathrm{baseline}$, where *X* is time, *Y* is a movement that starts at *Y*_0_ and decays down to the baseline, *Y*_0_ and the baseline have the same units as *Y*, and *K* is the rate constant equal to the reciprocal of the *X*-axis units. This model was fitted to the dataset using the least-squares regression method, and *p*-values were calculated for comparison of control versus predator-reared populations with the test. Survival was analysed using Mantel–Cox tests for the pairwise comparisons of the survival functions. In all cases, GraphPad Prism software was used to build graphs and calculate *p*-values. To check for a false-discovery rate, we performed the Benjamini–Hochberg test (electronic supplementary material, table S1). Numerical values for all tests, as well as other statistical parameters (d.f., chisq, *t*-statistics) can be found in electronic supplementary material, data.

## 3. Results

### (a) Predator stress induces a catabolic shift towards lipid oxidation

**Figure 1.**
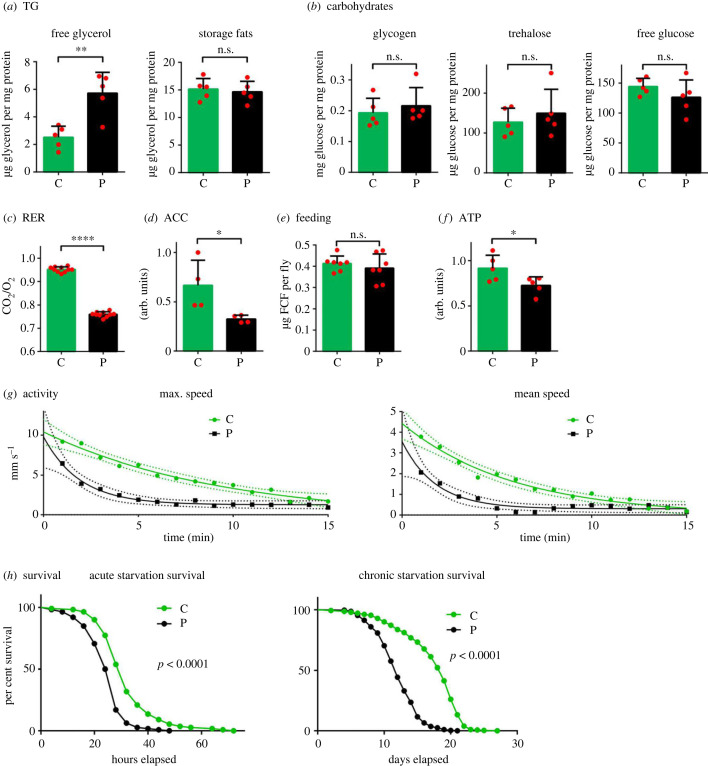
Effects of predator stress on the metabolism, locomotor activity, and survival in flies reared with spiders (predators) or without spiders (control). (*a*) Levels of free glycerol and storage fats. Relative but not absolute values of control flies have been published before [31]. TG, triglyceride. (*b*) Levels of carbohydrates: glycogen, trehalose, and free glucose. (*c*) Respiration exchange ratio (RER). (*d*) Amount of acetyl-CoA carboxylase (ACC) quantified against Ponceau S-stained total protein (for western blots, see electronic supplementary material figure S1). (*e*) Uptake of food containing 1% Blue FCF dye (FCF). (*f*) ATP concentration. (*g*) Nonlinear regression of maximum speed and mean speed measured across 15 min; test *p*-values for both cases are below 0.0001. Dots represent averages of 16 (control) and 23 (predator) experiments. The *p*-values of independent samples *t*-tests are 0.00017 for mean and 0.0047 for maximum speed. Dashed lines represent 95% confidence intervals. (*h*) Survival curves of flies kept on agar food (acute starvation, log-rank test *p* < 0.0001) and on 1% sucrose food (chronic starvation, log-rank test *p* < 0.0001). In all cases: *p < 0.05, **p < 0.01, ****p < 0.0001, n.s.—not significant. C—control, P—predator-reared.

Both carbohydrates and lipids, as key biochemical energy storage molecules, were measured in *Drosophila* Oregon strain flies reared with and without predatory spiders. While free glucose, its disaccharide trehalose, and polymeric form glycogen (*n* = 8) remained stable regardless of predator stress, triglycerides decreased, and free glycerol increased compared with controls (*n* = 10, figure 1*a,b*). This indicates increased utilization of lipids since lipolysis of triglycerides would provide free fatty acids for catabolism and simultaneously increase free glycerol concentration. Such specific loss of fat stores without any change in carbohydrate concentrations strongly indicates a shift in catabolism rather than inducing an overall starvation phenotype. Indeed, the RER (*n* = 20) of 0.76 in spider-reared flies supported this interpretation (figure 1*c*), indicating a firm reliance on a fatty acid breakdown in fuelling systemic ATP production.

### (b) Predator stress reduces overall energy levels

Even if catabolism is re-oriented towards fatty acid oxidation, carbohydrates can contribute to this through de novo lipid synthesis. However, the levels of the rate-limiting enzyme acetyl-CoA carboxylase (ACC) controlling this process were decreased in flies experiencing predator stress (*n* = 8, figure 1*d* and electronic supplementary material, figure S1). Increased feeding intensity (*n* = 14), a typical response to resource scarcity in *Drosophila*, was not found (figure 1*e*). Complete reliance on only one type of catabolic fuel source caused a 20% decrease in steady-state ATP levels (*n* = 10, figure 1*f*). Not compensating for diminished ATP production by increasing food uptake or lipid synthesis must come at the cost of lower metabolism. Spider-reared flies were indeed observed to have lower speed than controls in walking/climbing assays (*n* = 24 and 16, figure 1*g*). Similarly, these flies were less resistant to both acute (*n* = 220) and chronic (*n* = 274 and 275) starvation, exhibiting shorter survival in conditions of limited food resources (figure 1*e*).

### (c) Glucose uptake is inhibited

**Figure 2.**
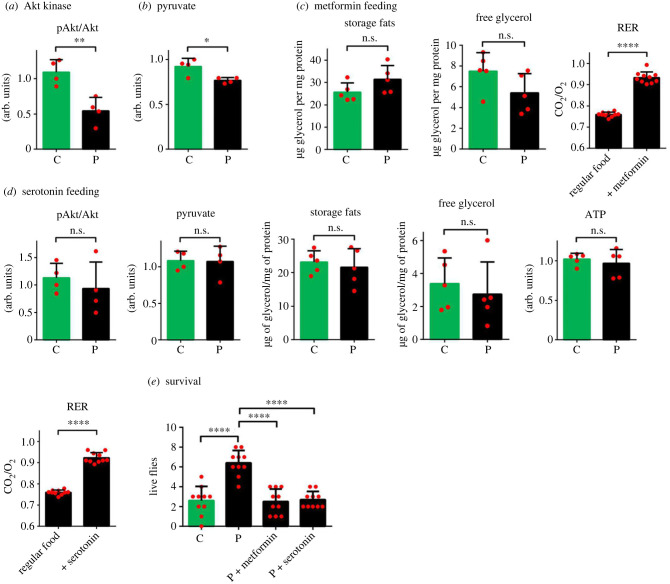
Effects of predator stress on metabolism/behaviour and pharmacological complementation in flies reared with spiders (predators) or without (control) spiders. (*a*) Phosphorylation of Akt kinase at Ser505. (*b*) Levels of pyruvate. (*c*) Effect of metformin feeding on storage fats, free glycerol, and predator-reared flies' respiration exchange ratio (RER). (*d*) Effects of serotonin feeding on Akt phosphorylation, pyruvate, storage fats, free glycerol, ATP and predator-reared flies’ RER. (*e*) Survival of predator-reared and control flies with or without feeding 5-hydroxytryptophan (5-HTP) or metformin after 12 h of incubation with spiders. For better comparison, RER data of predator-reared flies from figure 1*c* are re-used in (*c*,*d*). In all cases: *p < 0.05, **p < 0.01, ****p < 0.0001, n.s.—not significant. C—control, P—predator-reared.

The activity of Akt, a central regulator of the conserved glucose uptake mechanism, is dependent on the phosphorylation state of threonine at its kinase domain and serine residue in its hydrophobic motif (at position 505 in *Drosophila* Akt), which was found to be significantly decreased in spider-reared flies (*n* = 8, figure 2*a* and electronic supplementary material, figure S1). This indicates reduced glucose transport, depriving glycolysis of its substrate and decreasing its end-product pyruvate (*n* = 4, figure 2*b*). Administering metformin, an anti-diabetic drug that facilitates glucose uptake in both humans and flies [32,33], restored the normal balance in the flies' carbohydrate/lipid usage (*n* = 10) and increased their RER (*n* = 20) to normal value (figure 2*c*).

### (d) Serotonin complements metabolic dysfunction

The responses to external stimuli leading to different stress conditions are often mediated by changes in neurotransmitter levels. Serotonin dysregulation has been specifically associated with neurological stress that can cause several types of disorders in humans. In fact, *w^1118^* strain flies with a mutation in the *white* gene and severely reduced serotonin levels compared with red-eyed strains [34,35] displayed a much stronger metabolic shift (*n* = 10, electronic supplementary material, figure S2). This could mean that serotonin mediates the effects of predator stress downstream from other parts of fly metabolism. We therefore asked whether elevated serotonin can alleviate predator-induced metabolic impairment. We fed flies with elevated concentrations of the serotonin precursor 5-HTP and analysed its effects on Akt phosphorylation (*n* = 8), pyruvate (*n* = 8), triglycerides (*n* = 10), free glycerol (*n* = 10), ATP (*n* = 10) and RER (*n* = 20) (figure 2*d*). In all cases, external administration of serotonin precursor restored these parameters in spider-reared flies to control levels, suggesting that supporting serotonin synthesis is sufficient for countering these metabolic alterations.

### (e) Survival of flies under predation

The aforementioned changes in metabolism and decreased locomotor activity led us to ask whether predator presence affects the survival of flies. We housed flies together with predatory spiders (10 male flies and 1 spider per group; 10 experimental groups in total) in a closed space and observed the survivability of flies over 12 h. Surprisingly, there was an apparent increase in the survivability of predator-reared flies over control flies (figure 2*e*). Remarkably, feeding metformin and a precursor of serotonin that reversed metabolic defects also decreased the survival of predator-reared flies to levels observed in the control group (*n* = 20). This demonstrates that the increased survival of flies in response to predator presence comes at the cost of metabolic health.

## 4. Discussion

The effects predators have on prey are not limited to the death of prey individuals, but can induce a lasting condition of fear in the prey that survive in the presence of predators. As a result, prey often respond to predators in their environment by altering their morphological and physiological phenotypes during development [30,36–39]. Although these changes facilitate survival by improving escape abilities [40,41], predators may have enduring costly effects on prey individuals [42–44]. For example, predator-induced fear is one of the most common stressors employed in animal model studies of post-traumatic stress disorder [43]. This research has gained scientific interest because of the relevance of psychological stress in causing clinical depression and other metabolic disorders, such as type 2 diabetes, in humans. Although the underlying mechanism has remained unclear, increased serum glucocorticoid concentrations and catecholamine release are commonly associated with the development of insulin resistance [45]. Our results align with these findings by showing that *Drosophila* reared with predators develop a diabetes-like biochemical phenotype characterized by an inability to metabolize glucose, forcing a shift to triglyceride consumption. This is caused by a decreased activity of Akt kinase, a central regulatory kinase that has a major role in controlling glucose uptake. This protein facilitates a highly conserved glucose transport mechanism (e.g. via GLUT4-dependent pathway in muscles), and defects in this pathway are therefore closely associated with the development of diabetes [46]. Improving glucose transport using metformin, which has similar effects in flies to those in humans [33], restored the original metabolic balance in flies grown with predators.

Predator presence eventually changes the quality of the environment and affects the survival strategies of prey. While *Drosophila* flies rely on visual and olfactory cues for detecting predators such as spiders and mantises, it is currently unclear to what extent flies use separate sensory systems in different environmental conditions [30,47]. However, they do have a highly developed olfactory system that allows them to live for generations in complete darkness [48]. This sensory system is sufficient by itself to detect the presence of spiders, and even exposure to spider odours can elicit metabolic and developmental changes [30,49]. Regarding *w^1118^* flies, it is also unclear whether they can use vision to detect predators, as they may have poor visual acuity [50]. However, *w^1118^* flies easily chose walking corridors when tested in the Y-maze experiments [51], suggesting *w^1118^* flies actively rely on vision while exploring their environment.

Although a number of studies have described how chronic stress can have enduring effects on metabolism and behaviour [43,52,53], the connection between neural chemistry and metabolism has remained unclear. Our finding that supporting serotonin synthesis antagonizes the described metabolic effects suggests a central role for serotonin in such biochemical communication. Serotonin has multiple biological functions: regulating courtship behaviour [54], affecting spatial memory [55] and olfactory learning [56], influencing phototactic behaviour [49,57], and affecting turning behaviour [58]. Furthermore, it participates in several pathways that overlap with the roles of other neurotransmitters, such as dopamine and octopamine (norepinephrine homologue in *Drosophila*). Owing to the variety of serotonin's roles in neural circuits, which are at least partially redundant, its effect on metabolism can be caused by a number of different mechanisms. One plausible explanation could be related to the observed interconnection between serotonergic and insulin-producing nervous systems. In *Drosophila*, serotonergic neurons are closely apposed with insulin-producing neurons, and these two neuronal systems communicate [59]. They control insulin signalling and, if defective, serotonin and insulin accumulate together, with suppressed peripheral insulin sensitivity. In humans, elevating serotonin has beneficial effects on metabolic balance, improving insulin sensitivity and glucose homeostasis [60]. This effect is relayed through serotonylation of the small GTPase Rab4, which elicits beneficial effects on glucose uptake, thus representing a convergence point between serotonin and insulin signalling. Since serotonin is decreased in human psychological disorders resembling the effects of predator stress, it is tempting to speculate a linear relationship between the metabolic reprogramming described here and serotonin levels. Tentative support for this hypothesis comes from a quantitatively stronger metabolic shift in the serotonin-depleted *w^1118^* strain. However, serotonergic upregulation caused by the exogenous administration of serotonin might also elicit the observed reversion of metabolic changes.

Systemic effects on catabolism in predator-stressed flies resembled the effect of glucocorticoids in humans, a group of hormones released in response to stress conditions through activation of the HPA axis. These hormones antagonize the function of insulin by inhibiting the uptake of glucose in muscles and adipose tissue. They also downregulate glycolysis, inducing lipolysis and hepatic gluconeogenesis [20]. This mobilizes and reroutes energy reserves for specific tasks, e.g. increasing blood glucose levels to prepare the organism for a ‘flight-or-fight’ response.

*Drosophila* has no apparent neuroanatomical homologue of the mammalian HPA axis nor the same glucocorticoid hormones as humans. However, it has a central steroid hormone ecdysone, converted into 20-hydroxyecdysone (20HE) in haemolymph after its release. Best known for its role in inducing larval moults and metamorphosis [61], it also regulates metabolism by suppressing glucose use. Binding with its receptor (EcR) induces this protein's translocation to the nucleus, where it represses the transcription of genes central to glucose utilization [62]. This is antagonistic to the function of an oestrogen-like receptor (ERR) recently described as a receptor for glucocorticoids in *Drosophila*, suggesting an interplay with other steroid hormones in this organism [63,64]. The effects of 20HE are very similar to the deletion of ERR, which blocks the use of carbohydrates as a fuel source, leading to a shift towards lipid oxidation and depleting triglyceride reserves [64]. Furthermore, 20HE acts as a stress hormone in flies, upregulated in response to adverse environmental conditions and stressful social interactions [65].

Intriguingly, we found that predator stress enhances the survival of spider-reared flies in the adult stage when kept together with the spiders. This effect correlated precisely with metabolic reprogramming since the administration of metformin and serotonin precursor reverted the survival advantage to control levels. This indicates that this metabolic reprogramming is adaptive and provides a clear survival benefit at the expense of reduced metabolic fitness. One explanation for this finding is associated with the speed of movement and overall locomotor activity of the flies. Another possibility is linked to the glucocorticoid stress effect on memory. Stress-induced glucocorticoid release enhances memory consolidation and long-term memory in humans [66]. The effect is the same in flies, with ecdysone having a clear beneficial impact on long-term memory formation [65,67]. It is believed that these effects of glucocorticoids are linked to the conservation of glucose for neural tissue function, which is a primary carbon source. This adaptation fuels increased neural activity, especially learning and memory [68]. Brains are metabolically costly organs, as is the process of creating new memories (e.g. via increased synaptic connections) [69]. In fact, elevated carbohydrate uptake in humans and animals, including *Drosophila*, has an apparent memory-enhancing effect, especially for long-term memory [70–74].

In the aggregate, the results of this study allow us to propose a model explaining how chronic psychological stress, such as predator stress, induces metabolic disorders. Shunting glucose away from catabolically active tissues like muscle to be consumed by neurons is likely an adaptation to create memories and prepare for similar stressful conditions in the future. However, when stress persists and leads to chronic activation of the HPA axis and sustained glucocorticoid release, it will impair normal glucose metabolism and permanently shift systemic catabolism towards lipid oxidation, preventing the use of carbohydrates. Such loss of metabolic flexibility, especially in animals that use carbohydrates as the main form of energy source, will inflict fitness costs, leading to decreased ATP production and downstream effects on resistance to nutritional scarcity and locomotor activity. Therefore, this chronic activation of a mechanism that provides short-term benefits will cause decreased fitness if stress persists. This is supported by the observation that chronic activation of ecdysone signalling, although beneficial for an immediate response, can cause negative long-term effects [75].

The results of this study suggest that although the diabetes-like phenotype induced by predator presence reduces general health, it might be beneficial for survival. The insulin-producing system in *Drosophila* and other invertebrates differs to some extent from that of vertebrates, including humans. *Drosophila* flies have eight insulin-like peptides [76], which likely have different and partially overlapping roles in metabolism regulation [77]. This shows that insects may have numerous ligands for one receptor, while mammals have receptors with somewhat redundant functions but a restricted number of ligands. Also, while the effect of extra 5-HTP in increasing serotonin is straightforward, it might affect concentrations of another neurotransmitter. Tryptophan is a precursor of biopterin [78], a cofactor associated with serotonin and dopamine synthesis. While the metabolic shift in serotonin-depleted *w^1118^* flies compared with the Oregon strain provides tentative support for decreased serotonin concentration in response to predator stress, neuron-specific measurements are required to fully understand the mechanism underlying this hypothetically adaptive metabolic shift.

Finally, metabolic disorders are often associated with the impairment and loss of dopaminergic function [79]. Predator-induced stress affects the levels of brain dopamine, which are decreased in rats exposed to predator stress [80,81]. Since the *w^1118^* strain has reduced dopaminergic activity, the interconnected serotonin and dopamine pathways should be studied simultaneously in predator-induced stress. These numerous aspects must be considered to fully understand the role of stress in the development of metabolic phenotypes and similarities/dissimilarities of stress perception in humans and *Drosophila* [82].

## Data Availability

All data with individual numerical data points are available in the figures and in electronic supplementary material, data file [83]. The 'Material and methods' section contains all information necessary for replication of experiments.
